# Significance analysis of microarray transcript levels in time series experiments

**DOI:** 10.1186/1471-2105-8-S1-S10

**Published:** 2007-03-08

**Authors:** Barbara Di Camillo, Gianna Toffolo, Sreekumaran K Nair, Laura J Greenlund, Claudio Cobelli

**Affiliations:** 1 Information Engineering Department, University of Padova, 35131 Padova, Italy; 2 Endocrinology Division, Mayo Clinic, Rochester, Minnesota 55905, USA

## Abstract

**Background:**

Microarray time series studies are essential to understand the dynamics of molecular events. In order to limit the analysis to those genes that change expression over time, a first necessary step is to select differentially expressed transcripts. A variety of methods have been proposed to this purpose; however, these methods are seldom applicable in practice since they require a large number of replicates, often available only for a limited number of samples. In this data-poor context, we evaluate the performance of three selection methods, using synthetic data, over a range of experimental conditions. Application to real data is also discussed.

**Results:**

Three methods are considered, to assess differentially expressed genes in data-poor conditions. Method 1 uses a threshold on individual samples based on a model of the experimental error. Method 2 calculates the area of the region bounded by the time series expression profiles, and considers the gene differentially expressed if the area exceeds a threshold based on a model of the experimental error. These two methods are compared to Method 3, recently proposed in the literature, which exploits splines fit to compare time series profiles. Application of the three methods to synthetic data indicates that Method 2 outperforms the other two both in Precision and Recall when short time series are analyzed, while Method 3 outperforms the other two for long time series.

**Conclusion:**

These results help to address the choice of the algorithm to be used in data-poor time series expression study, depending on the length of the time series.

## Background

A crucial issue in genomic studies is the elucidation of how genes change expression and interact as a consequence of external/internal stimuli such as an illness, drug administration, hormone stimuli, etc. Microarray technology makes it possible to monitor simultaneously a large number of gene transcripts through a series of different experimental conditions. In particular, microarray time series studies are essential to understand the dynamics of biological events at the molecular level.

A first necessary step in order to limit the analysis to those genes that change expression over time is to select differentially expressed transcripts. Selection methods proposed in the literature usually deal with the comparison of static (e.g. no treatment vs treatment) rather than dynamic conditions, and are based on statistical tests [1,2]. These methods test the significance of the differential expression gene by gene. At least two replicates for each of the conditions to be tested are necessary, but a higher number is required to have reliable results. In time series experiments, in which gene expression is monitored over time, it is necessary to test differential expression at different sampling times. ANOVA or ANOVA based procedures [3] have been proposed to this purpose. However, since in time series experiments replicates are often available only for a limited number of samples, ANOVA tests are seldom applicable. For this reason, differentially expressed genes in time series experiments are often selected using an empirical constant fold change threshold [4]. This is far from ideal, since it is based on an arbitrary choice (e.g. FC = 3), which does not take into account the characteristics of the measurement error.

When the number of the replicates is not sufficient to apply traditional statistical tests, alternative methods need to be applied. Two methods based on a fit of the time series were recently proposed in the literature [5,6]. These methods fit the time series expression profiles using respectively polynomials and splines. Comparison between time series is based respectively on model parameters and goodness of fit. Both methods are really general and do not require any replicates; however, it is not clear the role of the number of available samples on their performance.

Here we propose Methods 1 and 2 able to select differentially expressed gene profiles in data-poor conditions, based on a model of the experimental error. Their performance is investigated in comparison to method [6] (Method 3 in the following), based on splines fit, using synthetic time series of different length. Finally, a case study on insulin treated muscle cells is presented to better appreciate the implementation aspects of Methods 1 and 2.

## Methods

### Selection strategy

Let's call x^T^(t_k_) and x^C^(t_k_) the log-expression measurements in treated (T) and control (C) cultures, available for a generic gene X at time sample t_k _(k = 1, ..., M, with M number of time samples). Log expression measurement are used, as in [7], because the signal is considered proportional to the log of the measurements, the error is considered log-additive, and the large range of expression intensities makes the log-expression practical.

The rationale adopted to label a gene X as differentially expressed in condition T vs C is described in details for methods 1 and 2 and is briefly reviewed for Method 3, since we refer to [6] for further details.

#### Method 1

The deviation of expression of gene X in T and C is calculated for each sample t_k _as:

d(t_k_) = x^T^(t_k_) - x^C^(t_k_)     (1)

The gene is considered differentially expressed in T vs C if |d(t_k_)| exceeds a threshold *θ*_d _in at least one sampling time t_k _(k = 1, ..., M):

|d(t_k_)| >*θ*_d _    (2)

where *θ*_d _is determined in correspondence to a significance level *α*, based on the null hypothesis distribution of d(t_k_). This distribution is modeled from d(t_k_) values calculated by Equation 1, with x^T^(t_k_) and x^C^(t_k_) measured on experimental replicates (see below), which provide a situation where genes are not differentially expressed, so that the null hypothesis is verified. Therefore, replicates of at least one time sample are necessary, to apply Method 1.

#### Method 2

**Figure 1:**
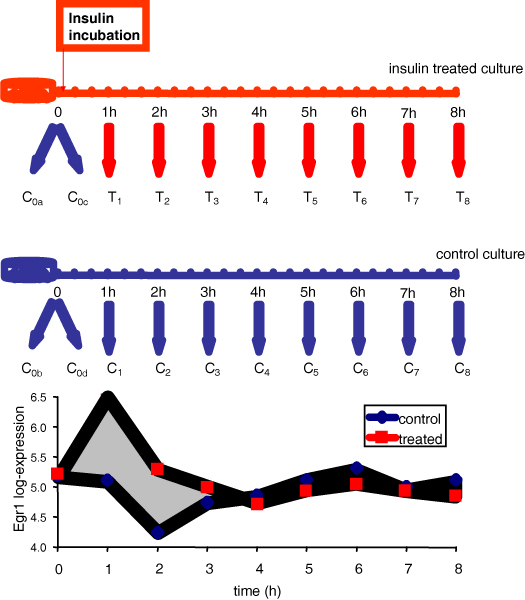
**Experiment outline**. The case study provides a typical example of experimental design in time series gene expression studies. Samples are collected from an insulin treated and a control culture. The expression level measured in treated and control culture for a single probe-set (corresponding to "Early growth response 1" gene) is shown in the lower part of the Figure. The area A bounded by the two expression profiles T and C is coloured in gray.

The area A bounded by the two expression profiles T and C (Figure 1) is calculated for each gene X as the sum of the contributions of partial areas from consecutive pairs of samples:

A=∑k=1M−1Ak     (3)
 MathType@MTEF@5@5@+=feaafiart1ev1aaatCvAUfKttLearuWrP9MDH5MBPbIqV92AaeXatLxBI9gBaebbnrfifHhDYfgasaacH8akY=wiFfYdH8Gipec8Eeeu0xXdbba9frFj0=OqFfea0dXdd9vqai=hGuQ8kuc9pgc9s8qqaq=dirpe0xb9q8qiLsFr0=vr0=vr0dc8meaabaqaciaacaGaaeqabaqabeGadaaakeaacqqGbbqqcqGH9aqpdaaeWbqaaiabbgeabnaaBaaaleaacqqGRbWAaeqaaaqaaiabbUgaRjabg2da9iabigdaXaqaaiabb2eanjabgkHiTiabigdaXaqdcqGHris5aOGaaCzcaiaaxMaadaqadaqaaiabiodaZaGaayjkaiaawMcaaaaa@3DA0@

Each contribution A_k _is calculated from the deviation of expression in T and C (Equation 1), as:

IF  sign(d(tk+1))=sign(d(tk))THEN  Ak=(d(tk+1)+d(tk))⋅(tk+1−tk)2ELSE  Ak=(d(tk+1)⋅d(tk+1)d(tk+1)+d(tk)+d(tk)⋅d(tk)d(tk+1)+d(tk))⋅(tk+1−tk)2     (4)
 MathType@MTEF@5@5@+=feaafiart1ev1aaatCvAUfKttLearuWrP9MDH5MBPbIqV92AaeXatLxBI9gBaebbnrfifHhDYfgasaacH8akY=wiFfYdH8Gipec8Eeeu0xXdbba9frFj0=OqFfea0dXdd9vqai=hGuQ8kuc9pgc9s8qqaq=dirpe0xb9q8qiLsFr0=vr0=vr0dc8meaabaqaciaacaGaaeqabaqabeGadaaakeaafaqaaeWabaaabaGaeeysaKKaeeOrayKaeeiiaaIaeeiiaaIaee4CamNaeeyAaKMaee4zaCMaeeOBa4MaeiikaGIaeeizaqMaeiikaGIaeeiDaq3aaSbaaSqaaiabbUgaRjabgUcaRiabigdaXaqabaGccqGGPaqkcqGGPaqkcqGH9aqpcqqGZbWCcqqGPbqAcqqGNbWzcqqGUbGBcqGGOaakcqqGKbazcqGGOaakcqqG0baDdaWgaaWcbaGaee4AaSgabeaakiabcMcaPiabcMcaPaqaaiabbsfaujabbIeaijabbweafjabb6eaojabbccaGiabbccaGiabbgeabnaaBaaaleaacqqGRbWAaeqaaOGaeyypa0ZaaSaaaeaacqGGOaakcqqGKbazcqGGOaakcqqG0baDdaWgaaWcbaGaee4AaSMaey4kaSIaeGymaedabeaakiabcMcaPiabgUcaRiabbsgaKjabcIcaOiabbsha0naaBaaaleaacqqGRbWAaeqaaOGaeiykaKIaeiykaKIaeyyXICTaeiikaGIaeeiDaq3aaSbaaSqaaiabbUgaRjabgUcaRiabigdaXaqabaGccqGHsislcqqG0baDdaWgaaWcbaGaee4AaSgabeaakiabcMcaPaqaaiabikdaYaaaaeaacqqGfbqrcqqGmbatcqqGtbWucqqGfbqrcqqGGaaicqqGGaaicqqGbbqqdaWgaaWcbaGaee4AaSgabeaakiabg2da9maalaaabaWaaeWaaeaacqqGKbazcqGGOaakcqqG0baDdaWgaaWcbaGaee4AaSMaey4kaSIaeGymaedabeaakiabcMcaPiabgwSixpaalaaabaGaeeizaqMaeiikaGIaeeiDaq3aaSbaaSqaaiabbUgaRjabgUcaRiabigdaXaqabaGccqGGPaqkaeaacqqGKbazcqGGOaakcqqG0baDdaWgaaWcbaGaee4AaSMaey4kaSIaeGymaedabeaakiabcMcaPiabgUcaRiabbsgaKjabcIcaOiabbsha0naaBaaaleaacqqGRbWAaeqaaOGaeiykaKcaaiabgUcaRiabbsgaKjabcIcaOiabbsha0naaBaaaleaacqqGRbWAaeqaaOGaeiykaKIaeyyXIC9aaSaaaeaacqqGKbazcqGGOaakcqqG0baDdaWgaaWcbaGaee4AaSgabeaakiabcMcaPaqaaiabbsgaKjabcIcaOiabbsha0naaBaaaleaacqqGRbWAcqGHRaWkcqaIXaqmaeqaaOGaeiykaKIaey4kaSIaeeizaqMaeiikaGIaeeiDaq3aaSbaaSqaaiabbUgaRbqabaGccqGGPaqkaaaacaGLOaGaayzkaaGaeyyXICTaeiikaGIaeeiDaq3aaSbaaSqaaiabbUgaRjabgUcaRiabigdaXaqabaGccqGHsislcqqG0baDdaWgaaWcbaGaee4AaSgabeaakiabcMcaPaqaaiabikdaYaaaaaGaaCzcaiaaxMaadaqadaqaaiabisda0aGaayjkaiaawMcaaaaa@D0EE@

The gene X is considered differentially expressed in T vs C if the following inequality holds:

A >*θ*_A _    (5)

where *θ*_A _is a threshold to be determined, in correspondence to a significance level *α*, based on the null hypothesis distribution of A, i.e. the distribution of A derived from experimental replicates (see below for its calculation).

#### Method 3

For each gene X, the expression profiles C and T are fitted using natural cubic splines. The null hypothesis is that both C and T time series share the same fit, the alternative hypothesis is that the fits are not equal. As a first step of the method, the same spline function is fitted simultaneously to the two profiles C and T and the sum of squares residuals SS^0 ^is calculated; then two different spline functions are fitted separately to time series C and T and the sum of squares residuals SS^1 ^is computed. To assess differentially expressed genes, the goodness of model fit under the null hypothesis is compared to that under the alternative hypothesis, by calculating a statistic F as:

F=SS0−SS1SS0     (6)
 MathType@MTEF@5@5@+=feaafiart1ev1aaatCvAUfKttLearuWrP9MDH5MBPbIqV92AaeXatLxBI9gBaebbnrfifHhDYfgasaacH8akY=wiFfYdH8Gipec8Eeeu0xXdbba9frFj0=OqFfea0dXdd9vqai=hGuQ8kuc9pgc9s8qqaq=dirpe0xb9q8qiLsFr0=vr0=vr0dc8meaabaqaciaacaGaaeqabaqabeGadaaakeaacqqGgbGrcqGH9aqpdaWcaaqaaiabbofatjabbofatnaaCaaaleqabaGaeGimaadaaOGaeyOeI0Iaee4uamLaee4uam1aaWbaaSqabeaacqaIXaqmaaaakeaacqqGtbWucqqGtbWudaahaaWcbeqaaiabicdaWaaaaaGccaWLjaGaaCzcamaabmaabaGaeGOnaydacaGLOaGaayzkaaaaaa@3E08@

Gene X is considered differentially expressed if the following inequality holds:

F >*θ*_F _    (7)

where *θ*_F _is a threshold to be determined, in correspondence to a significance level *α*, based on the null hypothesis distribution of F.

### Null hypothesis distribution model

Both Methods 1 and 2 are based on a threshold derived from the null hypothesis distribution of d(t_k_) and A, respectively, obtained using available replicates. We denote as "available replicates" replicates available for a subset of time samples (therefore we assume replicates available for at least one time sample).

Let's suppose two experimental replicates are available for a generic time t_k _and a generic experimental condition (T or C). By assuming a log-additive error model as in [7], the log-expression measurement of each gene X in replicates a and b, can be expressed as:

{xa=μ+εaxb=μ+εb     (8)
 MathType@MTEF@5@5@+=feaafiart1ev1aaatCvAUfKttLearuWrP9MDH5MBPbIqV92AaeXatLxBI9gBaebbnrfifHhDYfgasaacH8akY=wiFfYdH8Gipec8Eeeu0xXdbba9frFj0=OqFfea0dXdd9vqai=hGuQ8kuc9pgc9s8qqaq=dirpe0xb9q8qiLsFr0=vr0=vr0dc8meaabaqaciaacaGaaeqabaqabeGadaaakeaadaGabeqaauaabeqaceaaaeaacqqG4baEdaWgaaWcbaGaeeyyaegabeaakiabg2da9GGaciab=X7aTjabgUcaRiab=v7aLnaaBaaaleaacqqGHbqyaeqaaaGcbaGaeeiEaG3aaSbaaSqaaiabbkgaIbqabaGccqGH9aqpcqWF8oqBcqGHRaWkcqWF1oqzdaWgaaWcbaGaeeOyaigabeaaaaaakiaawUhaaiaaxMaacaWLjaWaaeWaaeaacqaI4aaoaiaawIcacaGLPaaaaaa@450F@

where *μ *represents the actual gene expression (unknown) and *ε*_a_, *ε*_b _are two realizations of the error variable *ε*, monitoring both technical and biological variability. The indices for condition and time t_k _are omitted here because we refer to pair of replicates available for a generic time sample t_k_.

#### Null hypothesis distribution of variable d

To quantify the deviation of expression d(t_k_) under the null hypothesis, Equation 1 is applied to available replicates as:

d^H0 ^= x_a _- x_b _= *ε*_a _- *ε*_b _    (9)

Different distribution models (t-Student distribution, bi-exponential distribution, and mixture models of N Gaussians, N = 1, ..., 6) are used to fit the set of d^H0 ^values obtained by applying Equation 9 to all genes and available replicates. The best model is selected based on the goodness of fit and the parameters precision, and is used as the null hypothesis distribution of d(t_k_) to determine *θ*_d _to be used in Equation 2.

To determine the threshold in correspondence to a significance level *α*, Method 1 uses a model to fit the observed statistics rather than using quantiles. The reason for this choice is that the lack of a sufficient number of observations from available replicates renders the determination of appropriate thresholds difficult when low significance levels are chosen, as often the case in microarray studies. If a sufficient number of replicates is available to guarantee a good threshold setting at the desired significance level *α*, it may be preferable to use quantiles.

#### Null hypothesis distribution of variable A

At least two replicates for each time sample would be necessary to derive A distribution under the null hypothesis from the data. Since we address selection of differentially expressed genes in data-poor condition, i.e. a sufficient number of replicates is not available, a Monte Carlo procedure is used to derive the null distribution of A. First, d^H0 ^distribution is derived from d(t_k_) values obtained from available replicates as described above. Then, B profiles of length M are sampled from d^H0 ^(here we used B = 10^4^) under the hypothesis that the error at different time samples is independent and identically distributed. Subsequently, B values of A^H0 ^are calculated from these profiles. Finally, different distribution models (Gamma, Log-normal, Weibull) are used to fit the entire set of A^H0 ^values and the best model is chosen based on goodness of fit and parameter precision. This model is used as the null hypothesis distribution of A to determine *θ*_A _to be used in Equation 5.

As for Method 1, a model is fitted to the observed statistics rather than using quantiles to determine the threshold in correspondence to a significance level *α*.

#### Null hypothesis distribution of variable F

The null distribution of F is obtained using bootstrap. See [6] for details.

### Intensity dependency of error

In Affymetrix chips it is well known that d^H0 ^has an intensity dependent distribution [8]. In particular, analysis of technical replicates of Affymetrix Human chip has shown that the standardized variable s^H0 ^(obtained dividing d^H0 ^by its standard deviation):

sH0=dH0SDdH0(x¯)=xa−xbSDdH0(x¯);x¯=xa+xb2     (10)
 MathType@MTEF@5@5@+=feaafiart1ev1aaatCvAUfKttLearuWrP9MDH5MBPbIqV92AaeXatLxBI9gBaebbnrfifHhDYfgasaacH8akY=wiFfYdH8Gipec8Eeeu0xXdbba9frFj0=OqFfea0dXdd9vqai=hGuQ8kuc9pgc9s8qqaq=dirpe0xb9q8qiLsFr0=vr0=vr0dc8meaabaqaciaacaGaaeqabaqabeGadaaakeaafaqabeqacaaabaGaee4Cam3aaWbaaSqabeaacqqGibascqaIWaamaaGccqGH9aqpdaWcaaqaaiabbsgaKnaaCaaaleqabaGaeeisaGKaeGimaadaaaGcbaGaee4uamLaeeiraq0aaSbaaSqaaiabbsgaKjabbIeaijabbcdaWaqabaGccqGGOaakcuqG4baEgaqeaiabcMcaPaaacqGH9aqpdaWcaaqaaiabbIha4naaBaaaleaacqqGHbqyaeqaaOGaeyOeI0IaeeiEaG3aaSbaaSqaaiabbkgaIbqabaaakeaacqqGtbWucqqGebardaWgaaWcbaGaeeizaqMaeeisaGKaeGimaadabeaakiabcIcaOiqbbIha4zaaraGaeiykaKcaaiabcUda7aqaaiqbbIha4zaaraGaeyypa0ZaaSaaaeaacqqG4baEdaWgaaWcbaGaeeyyaegabeaakiabgUcaRiabbIha4naaBaaaleaacqqGIbGyaeqaaaGcbaGaeGOmaidaaaaacaWLjaGaaCzcamaabmaabaGaeGymaeJaeGimaadacaGLOaGaayzkaaaaaa@5F0B@

has an intensity independent distribution [8]. Therefore, in case of data showing intensity dependency of the variable d^H0^, it is convenient to model s^H0 ^distribution, as indicated in the Additional File 1, to derive the threshold *θ*_d _to be used in Equation 2. Consistently, the values of d(t_k_) observed from the data are standardized before applying Equation 2, if Method 1 is used:

s(tk)=d(tk)SDdH0(x¯(tk));x¯(tk)=xT(tk)+xC(tk)2     (11)
 MathType@MTEF@5@5@+=feaafiart1ev1aaatCvAUfKttLearuWrP9MDH5MBPbIqV92AaeXatLxBI9gBaebbnrfifHhDYfgasaacH8akY=wiFfYdH8Gipec8Eeeu0xXdbba9frFj0=OqFfea0dXdd9vqai=hGuQ8kuc9pgc9s8qqaq=dirpe0xb9q8qiLsFr0=vr0=vr0dc8meaabaqaciaacaGaaeqabaqabeGadaaakeaafaqabeqacaaabaGaee4CamNaeiikaGIaeeiDaq3aaSbaaSqaaiabbUgaRbqabaGccqGGPaqkcqGH9aqpdaWcaaqaaiabbsgaKjabcIcaOiabbsha0naaBaaaleaacqqGRbWAaeqaaOGaeiykaKcabaGaee4uamLaeeiraq0aaSbaaSqaaiabbsgaKjabbIeaijabicdaWaqabaGccqGGOaakcuqG4baEgaqeaiabcIcaOiabbsha0naaBaaaleaacqqGRbWAaeqaaOGaeiykaKIaeiykaKcaaiabcUda7aqaaiqbbIha4zaaraGaeiikaGIaeeiDaq3aaSbaaSqaaiabbUgaRbqabaGccqGGPaqkaaGaeyypa0ZaaSaaaeaacqqG4baEdaahaaWcbeqaaiabbsfaubaakiabcIcaOiabbsha0naaBaaaleaacqqGRbWAaeqaaOGaeiykaKIaey4kaSIaeeiEaG3aaWbaaSqabeaacqqGdbWqaaGccqGGOaakcqqG0baDdaWgaaWcbaGaee4AaSgabeaakiabcMcaPaqaaiabikdaYaaacaWLjaGaaCzcamaabmaabaGaeGymaeJaeGymaedacaGLOaGaayzkaaaaaa@6586@

Analogously, if Method 2 is used on data showing intensity dependency of the variable d^H0^, A^H0 ^and *θ*_A _are derived using s^H0 ^and the values of A observed from the data (Equation 4) are calculated using s(t_k_) instead of d(t_k_).

### Threshold setting

Once the null hypothesis distribution of d, A and F are obtained, thresholds *θ*_d_, *θ*_A _and *θ*_F _are determined in correspondence to a significance level *α*. Rather than fixing it a priori, *α *can be optimized based on a variety of criteria aiming to control the family wise error rate (FWER) [9], or the false discovery rate (FDR) [10,11] or a compromise between false positive and false negative classification [12]. As an example, let's focus on a criterion based on the control of FDR, defined as the expected proportion of false positive classification (FP) among the number S_*α *_of genes selected as differentially expressed, using significance level *α*:

FDR=E[FPSα]     (12)
 MathType@MTEF@5@5@+=feaafiart1ev1aaatCvAUfKttLearuWrP9MDH5MBPbIqV92AaeXatLxBI9gBaebbnrfifHhDYfgasaacH8akY=wiFfYdH8Gipec8Eeeu0xXdbba9frFj0=OqFfea0dXdd9vqai=hGuQ8kuc9pgc9s8qqaq=dirpe0xb9q8qiLsFr0=vr0=vr0dc8meaabaqaciaacaGaaeqabaqabeGadaaakeaacqqGgbGrcqqGebarcqqGsbGucqGH9aqpcqqGfbqrdaWadaqaamaalaaabaGaeeOrayKaeeiuaafabaGaee4uam1aaSbaaSqaaGGaciab=f7aHbqabaaaaaGccaGLBbGaayzxaaGaaCzcaiaaxMaadaqadaqaaiabigdaXiabikdaYaGaayjkaiaawMcaaaaa@3E04@

In case of numerous sets as for microarrays, FDR is well approximated by

FDR≈E[FP]Sα     (13)
 MathType@MTEF@5@5@+=feaafiart1ev1aaatCvAUfKttLearuWrP9MDH5MBPbIqV92AaeXatLxBI9gBaebbnrfifHhDYfgasaacH8akY=wiFfYdH8Gipec8Eeeu0xXdbba9frFj0=OqFfea0dXdd9vqai=hGuQ8kuc9pgc9s8qqaq=dirpe0xb9q8qiLsFr0=vr0=vr0dc8meaabaqaciaacaGaaeqabaqabeGadaaakeaacqqGgbGrcqqGebarcqqGsbGucqGHijYUdaWcaaqaaiabbweafjabcUfaBjabdAeagjabdcfaqjabc2faDbqaaiabdofatnaaBaaaleaaiiGacqWFXoqyaeqaaaaakiaaxMaacaWLjaWaaeWaaeaacqaIXaqmcqaIZaWmaiaawIcacaGLPaaaaaa@3F45@

Calculating FDR requires the estimate of the expected number of false positives, obtained as the product of *α *by the number N_0 _of non differentially expressed genes:

E [FP] = N_0 _· *α *    (14)

N_0 _is unknown and is estimated using the bootstrap procedure described in [13].

FDR is calculated for a range of significance levels *α *and the significance level that guarantees the desired FDR is then used to select differentially expressed genes.

Since Method 1 applies M tests (corresponding to individual time points) for each gene, the significance level *α *for Method 1 is corrected by applying Šidák correction [14] in order to account for multiple testing.

### Simulation

Three different experimental conditions with a number of time samples M = 10, 30, 50 were simulated. 100 synthetic data sets were generated for each experimental condition, each consisting of 2000 profiles: 300 simulated as differentially expressed and the remaining as random noise. In both cases, the deviation of expression in T vs C was generated at each sampling time t_k _as standardized deviation:

s(t_k_) ~ N(*μ*_k_, *σ*^2^) ∀k = 1, ..., M     (15)

where *σ*^2 ^was set equal to 1 and *μ*_k _= 0 (k = 1, ..., M) for not differentially expressed genes; while, for differentially expressed genes, plausible profiles were obtained by modeling *μ*_k _as dependent on *μ*_k-1 _according to a first order Markov model (see Additional File 1 for details), with the only constraint of being greater than 1 (or lower than -1) for at least one time samples.

Samples k = 1 were generated twice for each gene, so as to provide replicates useful to apply Method 1 and 2. These replicates were included also in the analysis for method 3. Simulated data were used to test the performance of the methods in different experimental conditions. After the null hypothesis distributions of variables d(t_k_), A and F are modeled as described above, a significance level *α *had to be fixed to determine the confidence thresholds *θ*_d_, *θ*_A _and *θ*_F _to be used in Equations 2, 5 and 7 respectively. We compared the performance of the three methods across the entire range of significance level *α *by using Precision (true positives divided by the number of selected genes) vs Recall (true positives divided by the number of differentially expressed genes), and curves of observed false positives divided by the number of selected genes (observed FDR) vs number of selected genes. Moreover, we compared the average results obtained with the 3 methods by setting *α *in correspondence to a desired FDR of 0.05, in terms of number of selected genes and observed false positives divided by the number of selected genes. All measurements were averaged across the 100 simulations.

### Insulin case study

To better appreciate some characteristics of Methods 1 and 2 related to the experimental error modeling, the analysis of the null hypothesis distribution of the variables d(t_k_) and A (Equation 1 and 4) was applied to a real case study on rat muscle cells treated with insulin. The study was performed in vitro, on muscle L6 rat cell line. Cells were treated with insulin at time 0+, just after the collection of a first baseline sample at time 0; eight samples were harvested every hour during eight hours insulin stimulation. A control experiment was also performed in order to be able to distinguish between insulin effect and biological processes of different nature, which take place in the culture simultaneously to insulin induced processes (Figure 1). A total of twenty Affymetrix chips RG_U34A (monitoring 8.799 transcripts) were hybridized using four replicates of the basal sample, eight samples collected from the control culture, and eight samples collected from the treated culture. Standard Affymetrix MAS 5 software [15] was used for data pre-processing.

## Results

### Simulation

**Figure 2:**
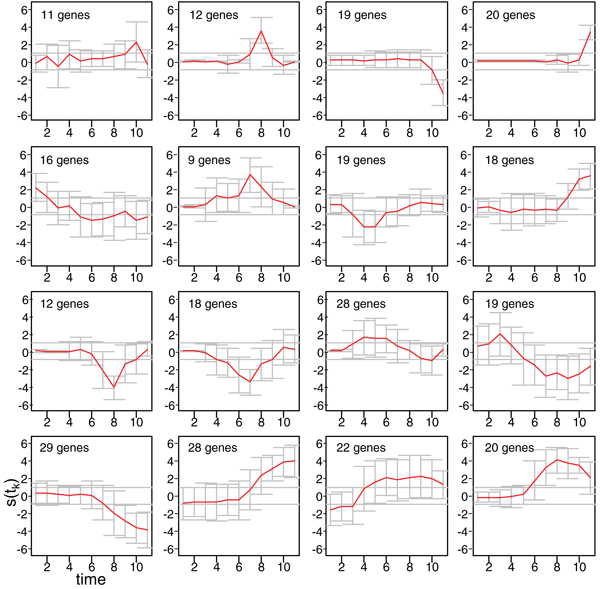
**Simulated data**. Standardized deviation s(t_k_) (k = 1, ..., M) between treated and control gene expression for 300 simulated differentially expressed profiles of a simulated data set (number of time samples M = 10) is shown as clusters obtained by using Self Organizing Maps and Pearson Correlation as similarity measure. For each cluster the average profile ± standard deviation and the number of genes are shown. The two horizontal lines correspond to the ± *σ *= ± 1 (Equation 15).

An example of simulated data with M = 10 is shown in Figure 2, as clusters obtained by using Self Organizing Maps [16] and Pearson Correlation as similarity measure on one of the simulated data-set (M = 10). The average profile is shown for each cluster, together with standard deviation bars; the number of genes for each cluster is also reported. A variety of profiles are represented, such as genes differentially expressed in one or few peaks apparently uncorrelated (1^st ^row panels), profiles that show characteristic bumps and waves of different length (2^nd ^and 3^rd ^row panels) or consistent trends along the time series, accompanied by few (3^rd ^row panels) or numerous (4^th ^row panels) time samples with average absolute value greater than the error standard deviation *σ *(Equation 15).

**Figure 3:**
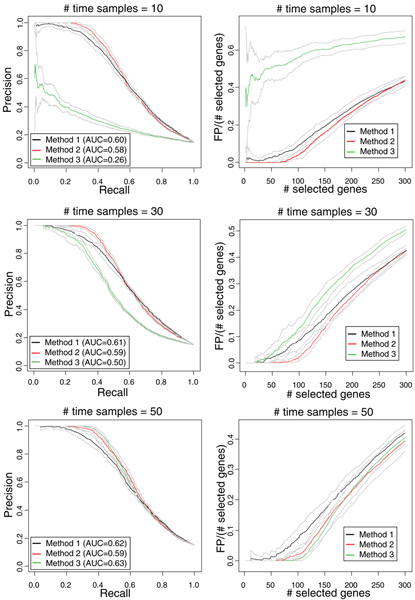
**Methods performance for simulated data**. Average Precision at different Recall intensities (left panels) and number of false positives divided by the number of selected genes for different number of selected genes (right panels) obtained on 100 simulated data sets, using methods 1, 2 and 3 on time series of 10 (upper left panel), 30 (upper right panel), and 50 (lower left panel) samples. AUCs are also reported for Precision vs Recall curves.

Results obtained by applying Methods 1, 2 and 3 to 100 simulated data sets are shown as average Precision at different ranges of Recall intensities in Figure 3, left panels. Average and standard deviations across the 100 simulations are reported. Method 2 slightly outperforms Method 1 for Precision higher than 0.6, since it provides higher values of both Precision and Recall in all the different sampling conditions. For values of Precision lower than 0.6, the two curves are almost superimposable. Both methods 1 and 2 work better than Method 3 for short time series (M = 10, M = 30), while for long time series (M = 50) the performance of the Methods 2 and 3 is similar, with Method 3 slightly superior to Methods 2. The areas under the curves (AUC) are also reported in Figure 3 (left panels). To better appreciate the ability of the three Methods to select differentially expressed genes at low FDR, Figure 3 (right panels) shows the number of observed false positives divided by the number of selected genes (observed FDR) for different numbers of selected genes in the range 0–300: Method 2 provides number of false positives divided by the number of selected genes always lower than that obtained with Method 1; Method 3 is slightly superior to Method 2 for M = 50, but inferior to both Methods 1 and 2 for M = 10 and M = 30. The performance of the three Methods with respect to each other is not affected by the number of differentially expressed genes (results not shown).

Table 1 shows the average number of selected genes and the proportion of false positives among the number of selected genes obtained by setting the confidence threshold according to an expected FDR of 0.05 estimated as described in section Methods (Equations 13, 14). Results confirm the ability of Method 2 to select more genes than the other two methods at a desired FDR, for M = 10 and M = 30. Moreover, the observed proportion of false positives among the number of selected genes is close to the estimated 0.05 for Method 2, while for Method 1 it is higher than the expected for M = 30 and M = 50. The performance of Method 3 is poor for short time series with M = 10, with an observed proportion of false positives among the number of selected genes much higher than 0.05 and a low number of selected genes (equal to 8). Method 3 outperforms Method 2 for M = 50, both in terms of number of selected genes and proportion of false positives on number of selected genes, thus confirming results shown in Figure 3 (right panels).

### Insulin case study

#### Error

**Figure 4:**
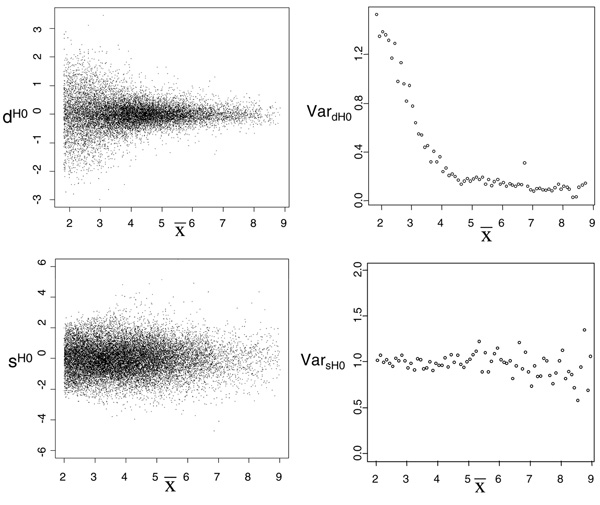
**Intensity dependency of the error**. Left panels: deviation of expression in Treated vs Control profiles calculated from pair of replicated measurements as a function of expression before (upper panel) and after standardization (lower panel). Right panels: average variance of d^H0 ^(upper panel) and s^H0 ^(lower panel) at different intensities. Dots represent mean variance of d^H0 ^(s^H0^) calculated in intervals of constant size (equal to 0.1).

**Figure 5:**
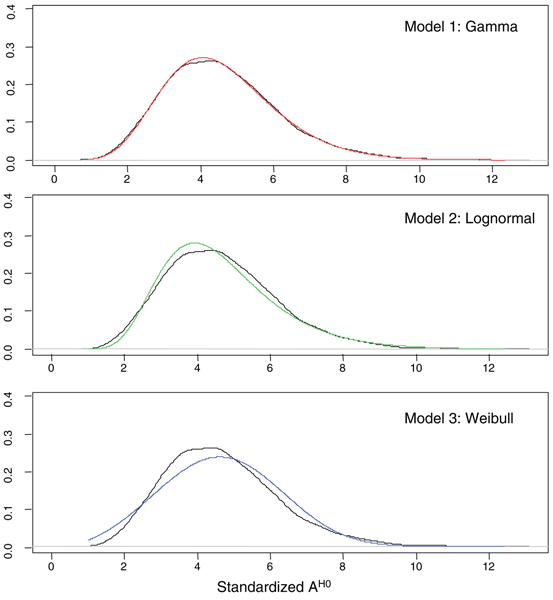
**Fit of A^H0 ^distribution**. A^H0 ^distributions (in black) fitted with a Gamma (upper panel) a Log-normal (central panel) and a Weibull distribution (lower panel).

In this section we present the estimate of the null hypothesis distribution of variable d(t_k_) (Equation 1) and variable A (Equation 4). Figure 4 shows the values of d^H0 ^(upper, left panel) (obtained by applying Equation 9 to the four available replicates) and its standardized value s^H0 ^(lower, left panel) plotted vs the average intensity of gene expression values (details on standardization step are given in Additional File 1). Figure 4 (right panels) shows the average variance of d^H0 ^and s^H0 ^vs the intensity range of gene expression discretized in intervals of constant size. Results confirm that while d^H0 ^has an intensity-dependent distribution, s^H0 ^has not [8] (results were insensitive to the bin size used to discretize the range of average intensity values). The entire set of s^H0 ^values was fitted using different distribution models (see Additional File 1 for details). The Gaussian mixture model with N = 2 was chosen as the best model. Model parameters and their precision are shown in Table 2. Monte Carlo procedure was then used to derive the null distribution of A: B profiles of length M = 8 were sampled from s^H0 ^distribution and B = 10000 values of A^H0 ^were calculated from these profiles. Three different distribution models (Gamma, Log-normal, Weibull) were used to fit the entire set of A^H0 ^values. The best fit for A^H0 ^was obtained with Gamma distribution (Figure 5 and Table 3).

#### Gene Selection

We applied the three Methods to the data, adopting the false discovery rate as criterion for threshold setting. Table 4 shows the number of probe-sets selected by each of the three methods for FDR equal to 0.001, 0.005, 0.01, 0.05 respectively. Results confirm those obtained by using simulation, i.e. the ability of Method 2 to select a higher number of differentially expressed genes at controlled FDR for short time series. Since focus here is methodology, biological results are not discussed further; confirmation studies and biological interpretation will be presented in a different article.

## Discussion

In this work we evaluated the performance of two selection methods here proposed to be applied in time series studies in data-poor conditions, i.e. when the number of available replicates does not make possible or practical the use of standard statistical methods. We also tested the two methods in comparison with a third method from the literature.

Method 1 compares samples time by time using a statistically based fold change threshold derived from a null hypothesis distribution of variable d(t_k_). To this purpose, replicates of at least one time sample are necessary. Since the threshold is derived based on the experimental variability, Method 1 accounts for the error characteristics, e.g. its intensity dependence; therefore, for example using Affymetrix chips, genes expressed at high intensities are not penalized with respect to genes at low intensities, which show a higher variability (Figure 4). Although Method 1 improves upon the use of a constant empirical fold change threshold, it considers time samples independently to each other, which is not a realistic assumption in time series studies. Method 2 calculates the area of the region bounded by the time series expression profiles to be compared and considers the gene differentially expressed if this area exceeds a threshold based on a model of the experimental error; therefore, besides accounting for the error distribution, it considers the entire expression profile and not single time samples. Also this method needs replicates for at least one time sample in order to derive the null hypothesis distribution. Both Methods 1 and 2 assumes as working hypothesis that the error at different time points is independent and identically distributed. This hypothesis, on our experience, is usually verified on real data (data not shown). However, for some experimental settings there may be a dependency of the error on time. In this latter case, it would be more appropriate to perform replicates at different time samples, covering the duration of the experiment, and use Method 1 with time dependent threshold settings. Methods 1 and 2 can be applied to compare time series from 2 different experimental conditions or a time series vs its baseline (e.g. time 0), by defining the deviation of expression of gene × for each sample t_k _(Equation 1) as the deviation between treated at time t_k _and the baseline. Moreover, if the sampling grid is different for the two time series, Method 2 can be easily generalized by generating A^H0 ^distribution from B time series with appropriate sampling grid.

Method 3 [6] uses natural cubic splines to fit time series expression data using a null hypothesis and an alternative hypothesis model, and performs a statistical test on the sum of squares residuals obtained using the two models to assess differential expression. It therefore, besides considering the entire expression profiles, implicitly considers time dependencies. Moreover, it does not need any experimental replicate, which makes it practical for a wide range of time series microarray experiments.

We tested the performance of the three methods using synthetic data to assess their validity over a range of experimental conditions, specifically the length of the analyzed time series. In the simulation, we used a Markov model to generate plausible data, accounting for the dependencies of time samples and not biased toward one of the methods. Taking for example *μ*_*k *_equal to an arbitrary constant for a random subset of time samples t_k _(k = 1, ..., M) in Equation 12, would not have accounted for time dependencies, and would have generated non realistic oscillation in the profiles, thus penalizing Method 3 which is based on a model fit, with respect to the other methods, in particular Method 1, which applies a threshold on each time sample. Moreover, simulated profiles (Figure 2) represent a variety of possible situations in time series expression, such as profiles characterized by one or few peaks, waves of different length or consistent trends along the time series.

Results on simulated data showed that Method 2 outperforms Method 1 independently from the length of the time series being analyzed, probably because, as Method 1 is based on single sample comparisons, it is particularly sensitive to random fluctuations due to the noise, thus resulting in a larger number of false positives. Method 2 constitutes an improvement with respect to Method 1 since the entire expression profile is considered simultaneously and this allows better distinguishing between consistent differences in expression profiles and random oscillations, thus resulting in a lower false negative rate. Method 3, as Method 2, considers the entire expression profile, but performs better than Method 2, only for long time series (M = 50).

Looking at the ability of the methods to classify profiles with particular characteristics, we observed that Method 1 works better than Method 2 to detect differentially expressed genes that show just one or two peaks as differentially expressed. On the opposite, Method 2 works better than Method 1 in detecting profiles characterized by waves of length greater than three samples, so as profiles that show a characteristic increasing/decreasing trend. Method 3, as Method 2, is better in detecting bumps and consistent trends in the profiles, than in detecting isolated peaks. However, it needs long time series (M = 50) to perform better than Method 2, probably because the fit are more reliable when performed on long time series; Method 3 was in fact proposed by the authors on real case studies with more than 40 samples. These results are certainly of interest to address the choice of the algorithm to be used in data-poor time series expression studies, depending on the availability of replicates and on the length of the time series.

## Conclusion

Microarray time series studies are essential to understand the dynamics of biological molecular events. In order to limit the analysis to those genes that change expression over time, it is necessary to select differentially expressed transcripts. Due to the high cost of microarrays, experiments are often performed without replication; therefore, traditional statistical methods can't be applied. Here we evaluate the performance of two selection methods applicable in data poor conditions, based on: a statistically based threshold on individual samples; a statistically based threshold to be applied on the area of the region bounded by the time series expression profiles to be compared.

Application on a real data set on insulin regulation on muscle cells, obtained using Affymetrix chips, revealed as the error analysis performed using Methods 1 and 2 may be useful to detect error characteristics such as intensity dependencies and to properly address these feature by standardization.

We evaluated Methods 1 and 2 performance using simulated data with a different number of available samples and compared these performance with those obtained using Method 3, based on a splines fit of time series profiles. The results outlines that the two error based Methods 1 and 2 work better than Method 3 with short time series experiments, while Method 3 works better than Methods 1 and 2 with long time series experiments. These results might help to optimize the choice of the algorithm to be used in different experimental conditions.

A preliminary version of Method 2, implemented in R, is available at [17].

## Authors' contributions

BDC conceived the study and performed data analysis under the guidance and supervision of GT. SKN was responsible for the overall coordination of microarray experiments realization. LJG performed microarray experiments. CC was responsible for the overall project coordination. All authors read and approved the final manuscript.

## Supplementary Material

Additional file 1 The .pdf document contains a detailed description of the procedure used to standardize the variable d^H0 ^so as to account for the error intensity dependent distribution, and a detailed description of the procedure used to simulate the data, based on a first order Markov Model. Click here for file

## Figures and Tables

**Table 1:** Results on simulated data for threshold setting based on desired FDR = 0.05.

	**Method 1**		**Method 2**		**Method 3**	
	
	# selected genes	# FP/# sel.genes	# selected genes	# FP/# sel.genes	# selected genes	# FP/# sel.genes
**M = 10**	**73 ** (17)	**0.045 ** (0.027)	**100 ** (8)	**0.040 ** (0.014)	**8 ** (10)	**0.304 ** (0.334)
**M = 30**	**91 ** (21)	**0.078 ** (0.045)	**120 ** (13)	**0.059 ** (0.028)	**83 ** (10)	**0.098 ** (0.046)
**M = 50**	**96 ** (16)	**0.068 ** (0.024)	**126 ** (12)	**0.058 ** (0.021)	**130 ** (12)	**0.053 ** (0.027)

All measurements were averaged across the 100 simulations; standard deviations are reported in parenthesis. Number of selected genes and number of false positives divided by the number of selected genes, obtained by applying the 3 methods on simulated data (setting *α *in correspondence to a desired FDR of 0.05).

**Table 2:** s^H0 ^distribution parameters. Parameters of the sum of two Gaussian mixture model for the distribution of s^H0^.

**Parameter**	**Estimate**	**Precision**
**a1**	0.64	0.03
**m1**	-0.07	0.01
**m2**	0.12	0.03
**sd1**	0.69	0.02
**sd2**	1.39	0.03

**Table 3:** A^H0 ^distribution parameters. Parameters of the Gamma distribution of A^H0^.

**Parameter**	**Estimate**	**Precision**
**shape**	8.9	0.1
**rate**	1.91	0.03

**Table 4:** Number of selected genes on real data for different threshold settings.

**FDR**	**# selected genes ** **Method 1**	**# selected genes ** **Method 2**	**# selected genes ** **Method 3**
**0.001**	216	50	6
**0.005**	279	87	14
**0.01**	320	100	15
**0.05**	451	156	46

Number of genes selected applying the 3 methods on real data (by setting *α *in correspondence to a desired FDR of 0.001, 0.005, 0.01, 0.05).
