# Supplementary material for: Significance analysis of microarray transcript levels in time series experiments
Source: BMC Bioinformatics. 2007 Mar 8;8(Suppl 1):S10. doi: 10.1186/1471-2105-8-S1-S10 (PMC1885839; doi:10.1186/1471-2105-8-S1-S10)
Supplement: Additional file 1 — The .pdf document contains a detailed description of the procedure used to standardize the variable dH0 so as to account for the error intensity dependent distribution, and a detailed description of the procedure used to simulate the data, based on a first order Markov Model. [file 1471-2105-8-S1-S10-S1.pdf]

Supplementary Material

Correction for intensity dependency of the error

Following [8], the procedure to obtain s^{H0} and its distribution was developed along the following steps.

1. For each gene X, the variable d^{H0} (Equations 8) and the average gene expression $\bar{x}$ are estimated from all available replicates.

2. The variance of d^{H0} is calculated. Replicates are not sufficient to calculate it reliably for each specific probe-set; therefore, the variance of d^{H0} is assumed gene-independent and evaluated using the overall probe-sets. The relation between d^{H0} and the average gene intensity $\bar{x}$ is made explicit performing the following steps:

- The range of average intensity values is quantized in intervals of constant size. Average intensities are calculated in each interval.
- For each of these intervals, the R genes with average expression $\bar{x}$ falling in the interval are considered. The corresponding d^{H0} values are used to calculate the variance of d^{H0} as:

$$\mathrm{Var}_{dH0} = \frac{1}{R-1} \cdot \sum_{r=1}^{R} \left(d_r^{H0} - m_{dH0}\right)^2 \qquad \text{(A1)}$$

  where m_{dH0} is the average of the R observed d_r^{H0} in the considered interval.
- Cubic splines are used to fit the variance of d^{H0} vs the average intensity of the gene expression.

3. The standard deviation of d^{H0} is derived as the square root of the variance Var_{dH0} estimated at the previous step and thus expressed as a function of the average intensity of the gene expression: $SD_{dH0}(\bar{x})$.

4. The standardized variable s^{H0} is calculated for each gene X and for each pair of replicates $x_{a,}$ x_b:

$$s^{H0} = \frac{d^{H0}}{SD_{dH0}(\bar{x})} = \frac{x_a - x_b}{SD_{dH0}(\bar{x})} \qquad \text{(A2)}$$

5. Different distribution models (t-Student distribution, bi-exponential distribution, and mixture models of N Gaussians, N=1, …, 6) are fitted to the entire set of s^{H0} values obtained by applying Equation (A3) to all genes and available replicates and the best model for s^{H0} distribution is chosen based on the goodness of fit and the parameters Precision

Data Simulation based on a first order Markov model

The standardized deviation of expression in T vs C is simulated at each sampling time t_k as:

$$s(t_k) \sim N(\mu_k, \sigma^2) \quad \forall\, k = 1, ..., M \qquad \text{(A3)}$$

where σ^2 is set equal to 1 and μ_k =0 (k=1, ..., M) for not differentially expressed genes; while, for differentially expressed genes, plausible profiles are obtained by modeling μ_k as dependent on μ_{k-1} according to a first order Markov model, according to the following procedure.

1. The range of the absolute value of variable $s(t_k)$ is quantized in 6 discrete values m_i (i=1, …, 6), corresponding to the average point of 6 equally spaced intervals between 0 and the maximum value of $s(t_k)$ (a part for i=1, for which the value $m_1=0$ is considered). In particular, here we used a maximum value of $s(t_k)$ equal to 6.
2. Different states $S_j(t_k)$ (j=1,...24) are defined for the variable $d(t_k)$ depending on its quantized value m_i, its sign (positive or negative), and the sign of its derivative.
3. The probability of transition among states (defining the probability of $d(t_k)$ being in state S_j given the state at the previous time point) is described by a Markov probability table of dimension 24 by 24.
4. Given the state $S_j(t_k)$ of the variable $d(t_k)$, the corresponding value of m_i is used as the value of μ_k in Equation A3. Note that, if i=1, m_i is set equal to 0 (state equivalent to a not differentially expressed time sample).

Instead of fixing arbitrary the probabilities of the table of transition among states, they were inferred based on observation on real data. In particular, a subset of profiles was used to calculate the frequency of occurrences of transition between states in subsequent time samples. The profiles were chosen as the union of the lists of the top 100 time series selected as differentially expressed by Methods 1, 2 and 3. To this purpose the three selection methods were applied to the experimental data presented in this work plus a second property data set on endothelial cells treated with insulin (data not shown).
